# Advanced serial analysis of the diaphragm surface EMG: insights into the effect of pressure support on the neuro-ventilatory response during the ICU stay

**DOI:** 10.1186/s13054-025-05424-5

**Published:** 2025-06-23

**Authors:** R. S. P. Warnaar, A. D. Cornet, A. Beishuizen, D. W. Donker, E. Oppersma

**Affiliations:** 1 Cardiovascular and Respiratory Physiology, Technical Medical Centre, University of Twente, Technohal 3184, P.O. Box 217, 7500 AE Enschede, The Netherlands; 2 Intensive Care Center, Medisch Spectrum Twente, Enschede, The Netherlands; 3 Intensive Care Center, University Medical Centre Utrecht, Utrecht, The Netherlands

**Keywords:** Mechanical ventilation, Respiratory failure, Respiratory surface electromyography, Neuro-ventilatory response

## Abstract

**Background:**

Ventilatory support levels in ICU patients should be tailored to both optimal gas exchange and respiratory muscle loading, as over- and underassistance may cause diaphragm dysfunction. The diaphragm’s capacity to overcome mechanical load and deliver ventilatory output is reflected by the patient’s neural respiratory drive (NRD), tidal volume (TV) and respiratory rate (RR). Surface electromyography of the diaphragm (sEMGdi) offers a continuous, non-invasive measure of NRD. We investigated the effect of pressure support (PS) level on the coupling of diaphragm electrical activity (sEAdi) and ventilatory output during the ICU stay.

**Methods:**

In clinically stable ICU patients (*N* = 17), four PS-levels were applied on alternate days, based on the clinical value (− 3, + 0, + 3, and + 6 cmH_2_O). sEAdi time-product (ETPdi) was calculated from high-quality sEAdi waveforms, using a novel, advanced signal analysis approach. The breath-by-breath correlation between ETPdi and TV was defined as neuro-ventilatory coupling (NVC), enabling quantification of the neuro-ventilatory response.

**Results:**

On group level (13 patients, 26 PS-trials), ETPdi and RR increased with decreasing PS-levels (2.4 and 1.6 percentage point (pp)/cmH_2_O), whereas TV decreased (2.5 pp/cmH_2_O). Longitudinal analysis (4 patients, 14 PS-trials) showed strengthened coupling between ETPdi and TV during weaning, reflected by an increase in median NVC from 3.4% (IQR 2.9) to 26.3% (IQR 21.7) between the first and last PS-trial.

**Conclusion:**

Advanced sEMGdi analysis allows for non-invasive quantification of NVC, reflecting the diaphragm’s capacity to overcome mechanical load. In patients approaching liberation from MV, increasing NVC indicates the shift from near-passive to active breathing. This study demonstrates the potential of NVC to inform tailoring of ventilatory support levels.

*Trial registration* number: Dutch Trial Register NL9654. Registered August 05, 2021.

**Supplementary Information:**

The online version contains supplementary material available at 10.1186/s13054-025-05424-5.

## Introduction

Patients receiving mechanical ventilation (MV) in the intensive care unit (ICU) are prone to develop diaphragm dysfunction [1], which is associated with longer MV durations and higher ICU mortality [2, 3]. Assisted modes of MV mitigate this phenomenon by enabling spontaneous respiratory efforts while unloading the patient’s respiratory muscles and assuring optimal gas exchange [4]. However, the level of ventilatory assistance should be adequately tailored, as both overassistance and underassistance may induce diaphragm dysfunction [5, 6]. In case of underassistance, the increased mechanical load induces a rise in neural respiratory drive (NRD), while the patient is unable to generate more ventilatory output [5–9]. Conversely, in overassistance, the provided support exceeds the ventilatory demands, leading to an attenuation of the patient’s respiratory drive, resulting in a near-passive breathing pattern [10, 11].

These neuro-ventilatory responses to over- and underassistance imply that the coupling between NRD and ventilatory output has the potential to reflect the capacity of the diaphragm to overcome mechanical load, which can inform tailoring the level of ventilatory support. In clinical practice, factors as disease severity, analgosedation, and ventilatory support all affect the relationship between NRD and ventilatory output [12], which is thus expected to change through the patient’s clinical course. Surface electromyography of the diaphragm (sEMGdi) offers a non-invasive measure of the NRD [13], thereby enabling its longitudinal bedside monitoring. We set out to investigate the course of NRD during assisted MV in the ICU and its relation to ventilatory output, level of pressure support and the patient’s clinical condition.

## Methods

### Study population

This research is part of a study on sEMGdi at varying ventilator settings [14]. The study protocol was approved by the medical ethical committee of Arnhem-Nijmegen, the Netherlands (CCMO-number NL75951.091.21) registered in the Dutch Trial Register (NL9654). A prospective cohort was included from the multidisciplinary ICU of Medisch Spectrum Twente, a tertiary referral hospital in Enschede, the Netherlands. Written informed consent was obtained from the patients’ legal representatives. Patients were eligible if aged ≥ 18 years, invasively ventilated for at least 48 h, and ventilated in pressure support mode (SPN-CPAP/PS, Drägerwerk AG & Co. KGaA, Lübeck, Germany) with a FiO_2_ ≤ 60%, a SpO_2_ ≥ 90%, and a Richmond Agitation and Sedation Scale (RASS) score ≤ 0. The RASS criterion was set to prevent measurements in agitated patients, as agitation could potentially be aggravated by varying the ventilator settings as dictated by the study protocol. This, in turn, would render crosstalk due to motor restlessness more likely. Exclusion criteria were a BMI > 30 kg/m^2^ at ICU admission, a persistent pneumothorax, a history of neuromuscular disease, or pregnancy.

### Data acquisition

sEMGdi was measured, as detailed before [14], at the eighth intercostal space in the right anterior axillary line with pre-gelled Ag/AgCl electrodes (3 M™ Red Dot™ 2560 electrodes, 3 M Deutschland GmbH, Neuss, Germany). An ECG lead was recorded from the sternal angle to the lower costal margin in the mid-axillary line for QRS complex detection. The electrodes were connected via actively shielded bipolar electrode cables (TMSi, Oldenzaal, the Netherlands) to a Mobi-6 device (TMSi, Oldenzaal, the Netherlands, 12.2 nV/bit, amplification factor: 19.5). The skin was cleansed with alcohol before electrode application. EMG and ECG signals were acquired at a sample rate of 2048 Hz using the TMSi MATLAB interface. Airway pressure (Paw), flow (F) and volume (V) tracings from the Dräger Infinity V500 ventilator (Drägerwerk AG & Co. KgaA, Lübeck, Germany) were acquired at 100 Hz through the ventilator’s RS232 interface.

### Study protocol

**Fig. 1:**
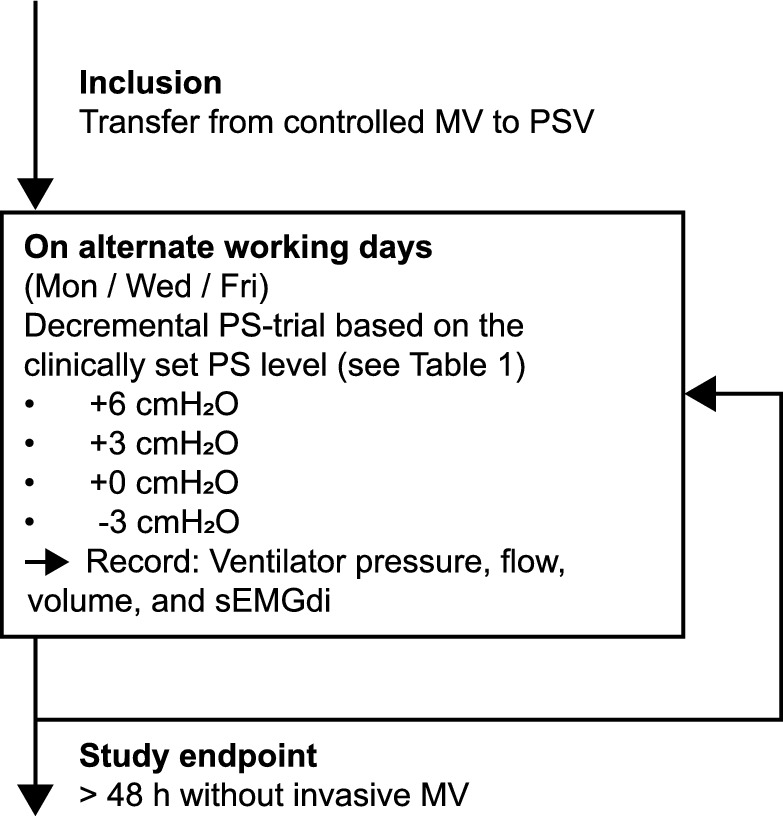
Study diagram

Measurements were performed on alternate working days as long as the patient still met the inclusion criteria (Fig. 1), and could be called off for medical reasons at the discretion of the attending physician. A decremental PS-trial was performed based on the clinically set PS-level, according to the protocol in Table 1. Other ventilator settings were maintained as dictated by routine clinical care. Each PS-level was applied for at least 5 min [15], extended up to 10 min in case of coughing or movement artefacts.

**Table 1:** Decremental PS-trial depending on clinically used PS-levels

		Study PS-trial levels (cmH_2_O)						
		21	18	15	12	9	6	3
**Clinically set PS-level (cmH**_**2**_**O)**	**≤ 8**				V	V	V	V
	**9–11**			V	V	V	V	
	**12–14**		V	V	V	V		
	**> 14**	V	V	V	V			

Bedside clinical data on the patient’s clinical status were extracted from the electronic medical record (Hix, Chipsoft B.V., Amsterdam, the Netherlands), including body temperature, body weight, fluid balance, ventilator settings, weaning strategy, need and type of analgosedation, and, if available, arterial blood gas (ABG) analysis.

### Offline signal pre-processing and parameter calculation

**Fig. 2:**
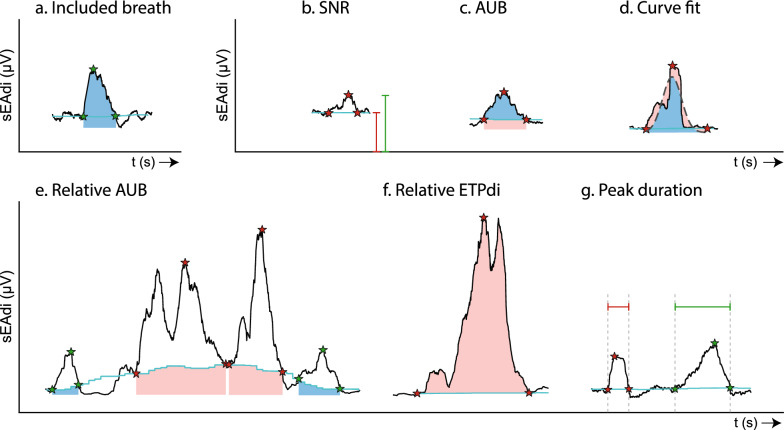
sEAdi quality aspects—a. Example of an included neural breath. ETPdi (blue) is calculated from baseline crossing to baseline crossing, including the area under the baseline (AUB). Quality assessment was based on previously defined quality criteria [14], evaluating b. signal-to-noise ratio (SNR), c. AUB, and peak morphology, and the additional criteria of e. AUB relative to the whole recording, f. ETPdi relative to the whole recording, and g. peak duration. The ratio of red to blue areas indicates peak quality, where more blue signifies better quality

sEMG signals were pre-processed using advanced data-analysis algorithms from the ReSurfEMG library [16]. Diaphragmatic activity peaks were automatically identified from the sEMGdi envelope: the surface electrical activity of the diaphragm (sEAdi). Breath-wise diaphragm activation, the neural breathing activity, was calculated as the area under the sEAdi curve including the area under the baseline (AUB) [14], resulting in an sEAdi time-product per breath (ETPdi, Fig. 2). High quality neural breaths were included after quality assessment according to the criteria illustrated in Fig. 2 [14]. Additionally, neural breaths were excluded if the sEAdi peak duration was less than 0.5 s, if the AUB exceeded four times the AUB upper quartile in that recording, or if the ETPdi exceeded ten times the ETPdi 95 th percentile, which were indicative of contamination by crosstalk.

Flow-based breaths were identified from the peaks in the volume tracing from the ventilator. Peaks were included if the tidal volume was larger than 20% of the 75-percentile of all detected volume peaks. The respiratory rate (RR) was calculated from the median inter-breath time (Tbreath). Flow-based breaths were linked to the nearest valid neural breath in the window from Tbreath/2 before to 0.5 s after the flow-based breath, as neural activation physiologically precedes or coincides with the flow-based breath.

### Data analysis

All PS-trials having at least 10% valid neural breaths at each PS-level, as compared to the flow-based respiratory rate, were included in the analysis. For the longitudinal analysis, each included neural breath was also matched to its associated flow-based breath. Data were analysed as median (interquartile range, IQR) unless stated otherwise.

The group-level effects of PS on normalised TV, RR, and ETPdi were examined using Generalised Estimating Equations (GEE) in SPSS (v. 28.0, IBM, Chicago, IL, United States). TV, RR, and ETPdi values were normalised relative to their median values at a PS of 12 cmH_2_O, as a PS of 12 cmH_2_O occurred in all PS-trials (Table 1). *P* < 0.05 was considered significant.

Post-hoc covariate analyses were performed in SPSS to explore which factors contributed to the variability in absolute ETPdi. Log-transformed median ETPdi at a PS-level of 12 cmH_2_O was modelled as function of the continuous covariates using linear regression. Dichotomous variables were tested using the Mann–Whitney U, and categorical variables by the Kruskal–Wallis test. Factors showing a significant correlation to ETPdi were included in a GEE, to evaluate whether the explanatory effect persisted when considering interdependency of measurements. *P* < 0.05 was considered significant during all post-hoc analysis steps.

The longitudinal development of NRD was explored qualitatively by plotting the linked neural breaths, i.e., ETPdi, and flow-based breaths, i.e., TV, per PS-level over the included PS-trials per patient. The R^2^ between the breath-by-breath ETPdi and TV was defined as the neuro-ventilatory coupling (NVC) and introduced as novel parameter to quantify the neuro-ventilatory response. Outliers in ETPdi and TV were excluded from this calculation if smaller than the first quartile −1.5 * IQR or larger than the third quartile + 1.5 * IQR.

## Results

A total of 43 PS-trials were performed in 17 patients (Table 2). After exclusion of invalid sEAdi peaks, 26 PS-trials (60%) from 13 patients were included for analysis. One PS-trial was excluded, because of extraordinary high flow trigger of 15 L/min. The included PS-trials per patient along with the pre-measurement SpO2, RASS-score, and MV settings can be found in Additional File 1. A detailed description of the in- and excluded neural breaths per PS-trial is provided in Additional File 2.

**Table 2:** Patient characteristics

Subject	Age (y)	Gender	BMI (kg/m^2^)	Reason of admission	Days of MV at inclusion (days)	Included PS-trials (N_incl_/N_tot_)
1	55	M	25	Pneumonia—COVID19	30	4/5
2	68	F	21	Sepsis—Abdominal	2	0/2
3	68	M	30	Pneumonia—COVID19	6	4/4
4	61	M	25	Pneumonia—COVID19, PE	12	3/4
5	61	F	18	Pneumonia—COVID19	5	1/1
6	76	M	23	Pneumonia—COVID19, PE	5	1/2
7	72	M	28	Surgical—Abdominal	6	1/3
8	55	F	29	Pneumonia—Unilateral Pneumococcus, Sepsis	4	0/1
9	49	M	25	Trauma—TBI	7	0/2
10	50	M	29	Trauma—TBI	15	2/2
11	52	F	23	COPD exacerbation	7	1/1
12	75	M	28	Surgical—Cardiothoracic (Valve replacement)	17	3/3
13	75	F	23	Surgical—Cardiothoracic (Morrow)	22	1/1
14	58	M	30	Trauma—Poly	20	1/1
15	74	M	29	Surgical—AAA	8	0/3
16	61	M	28	Sepsis—Lead endocarditis	6	1/1
17	78	M	28	Pneumonia—Legionella	3	3/7
						26/43

*MV* mechanical ventilation, *BMI* body mass index, *N*_*incl*_*/N*_*tot*_ number of included/total PS-trials, *PE* pulmonary embolism, *TBI* traumatic brain injury, *COPD* chronic obstructive pulmonary disease, *AAA* abdominal aortic aneurysm

### Group level effects of PS

**Fig. 3:**
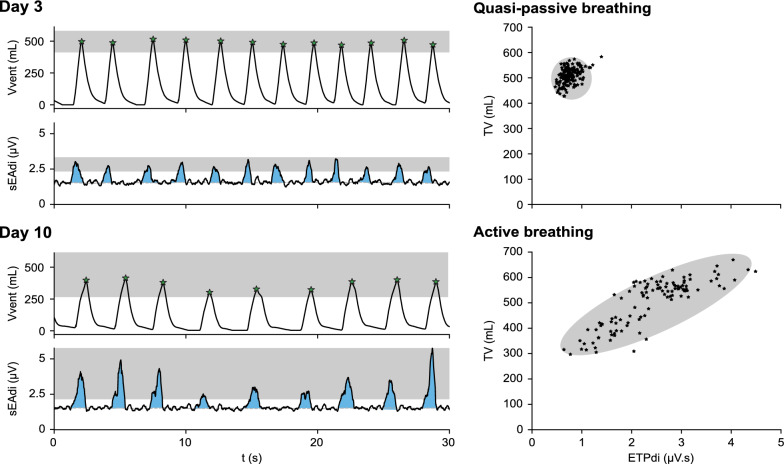
sEAdi and TV variability—sEAdi and volume waveforms for an individual patient at the same PS-level for the first and last included PS-trial, i.e. day 3 and day 10. sEAdi and TV variability increased from the first to the last PS-trial (left panels, shaded boxes), whereas the correlation between ETPdi and TV increased (right panels)

**Fig. 4:**
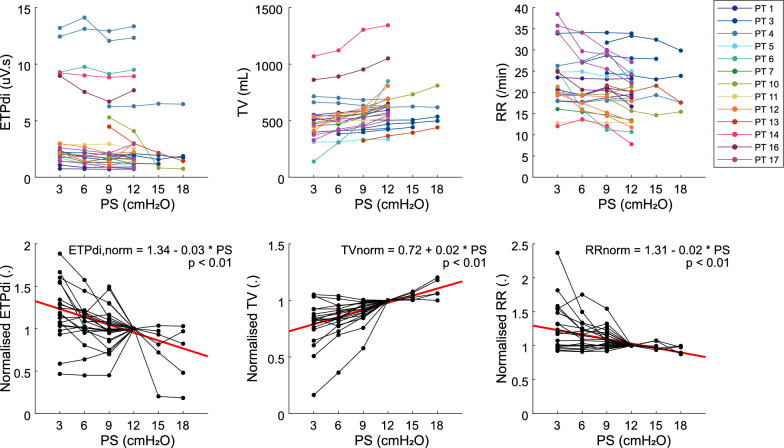
Effect of PS on absolute and normalised ETPdi, TV, and RR—Top: PS effect on absolute ETPdi, TV, and RR. Repeated PS-trials in one patient over separate days are indicated in the same colour. Bottom: PS effect on normalised ETPdi, TV, and RR. Black lines show the median values per PS-level within a PS-trial (one patient, one day). In red the GEE-models are shown. Normalised ETPdi, TV, and RR are unitless after normalisation to their median value at PS = 12 cmH_2_O

Absolute values of ETPdi, TV, and RR displayed large variability both between patients and PS-trials. Figure 3 illustrates the increased intra-patient variability in both sEAdi and TV between the first and last included PS-trial at the same PS-level. On group level, the sum of squares within a PS-trial (SSw) explained only 4%, 15% and 13% of the total variation (SStot) in ETPdi, TV, and RR (Fig. 4). When normalised to a PS-level of 12 cmH_2_O, the SSw/SStot increased to 54%, 46%, and 53%, respectively. Normalised ETPdi and RR showed a significant increase in response to a decreasing PS-level, whereas the TV significantly decreased (*p* < 0.01). ETPdi and RR increased by 2.4 and 1.6 percentage point (pp) per cmH_2_O of PS relative to a PS of 3 cmH_2_O, whereas TV decreased by 2.5 pp per cmH_2_O.

### Factors influencing absolute ETPdi

Univariate linear regression revealed four covariates that significantly correlated to the absolute ETPdi value at a PS-level of 12 cmH_2_O: TV, RR, body temperature, and the coefficient of variation (CoV) of the sEAdi moving baseline (Table 3). Only TV (*p* < 0.01) and body temperature (*p* = 0.02) remained significantly correlated to ETPdi at a PS-level of 12 cmH_2_O in a GEE using these parameters. The categorical factors—gender, bronchodilator use, need for analgosedation, and RASS-score—demonstrated no significant difference with regard to ETPdi at a PS-level of 12 cmH_2_O.

**Table 3:** Covariate analysis for ETPdi at a PS-level of 12 cmH_2_O

Continuous factors	R^2^†	*P*-value	
		Linear regression††	GEE††^§^
*Baseline characteristics*			
Age (y)	1%	0.69	
Height (cm)	3%	0.39	
Weight at admission (kg)	2%	0.49	
*Mechanical ventilation time*			
Day of invasive MV	4%	0.30	
Day of PSV	7%	0.19	
*Restlessness*			
CoV(sEAdi, baseline) (%)	19%	0.03*	0.08
*Fluid balance*			
Δ Fluid since inclusion (L)	9%	0.27	
Δ Weight since admission (kg)	4%	0.36	
ECG amplitude (µV)	1%	0.57	
*Arterial blood gas*			
pH	< 1%	0.78	
PaCO_2_ (kPa)	4%	0.41	
HCO3- (mmol/L)	9%	0.19	
PaO_2_ (kPa)	6%	0.29	
*Infectious status*			
Body temperature (℃)	16%	0.05*	0.02*
*Clinical ventilator settings*			
FiO2	4%	0.33	
PEEP (cmH_2_O)	5%	0.29	
Clinical PS (cmH_2_O)	0%	0.99	
*Ventilatory output*			
TV (mL)	31%	< 0.01**	< 0.01**
RR (/min)	15%	0.05*	0.35

Categorical factors (N_incl_/N_tot_)	$\overline{\text{ETPdi} }$ (IQR)	Mann–Whitney U / Kruskal–Wallis††	GEE††
*Gender*		0.88	
Male (23/37)	2.1 µV.s (4.9)		
Female (3/6)	2.5 µV.s (.)^§§^		
*Bronchodilators*		0.55	
Yes (2/5)	2.8 µV.s (.)^§§^		
No (24/38)	2.0 µV.s (4.5)		
*Analgosedation*		0.18	
Yes (10/20)	3.0 µV.s (6.8)		
No (16/23)	1.9 µV.s (1.6)		
*RASS-score*		0.56	
+ 1 (3/4)	1.7 uV.s (.)^§^		
0 (8/13)	2.0 uV.s (6.2)		
− 1 (4/4)	1.4 uV.s (1.8)		
− 2 (5/5)	2.9 uV.s (3.5)		
− 3 (0/5)	.^§§^		
− 4 (4/8)	5.6 uV.s (10.9)		
− 5 (3/4)	4.1 uV.s (.)^§§^		

*TV* tidal volume, *RR* respiratory rate, *CoV* coefficient of variation, *PaCO*_*2*_ arterial partial pressure of CO_2_, *PaO*_*2*_ arterial partial pressure of O_2_, *FiO*_*2*_ fraction inspired oxygen, *PEEP* positive end-expiratory pressure, *PS* pressure support, *PSV* pressure support ventilation, *N*_*incl*_*/N*_*tot*_ number of included/total PS-trials

*P < 0.05, **P < 0.01

*†* R^2^ determined from univariate linear regression, correlation to log(ETPdi)

*††* Covariate analysis based on log(ETPdi)

§ Only significant parameters in univariate regression are included in the GEE

§§ Could not be obtained from small number of datapoints

### Longitudinal neuro-ventilatory response

**Fig. 5:**
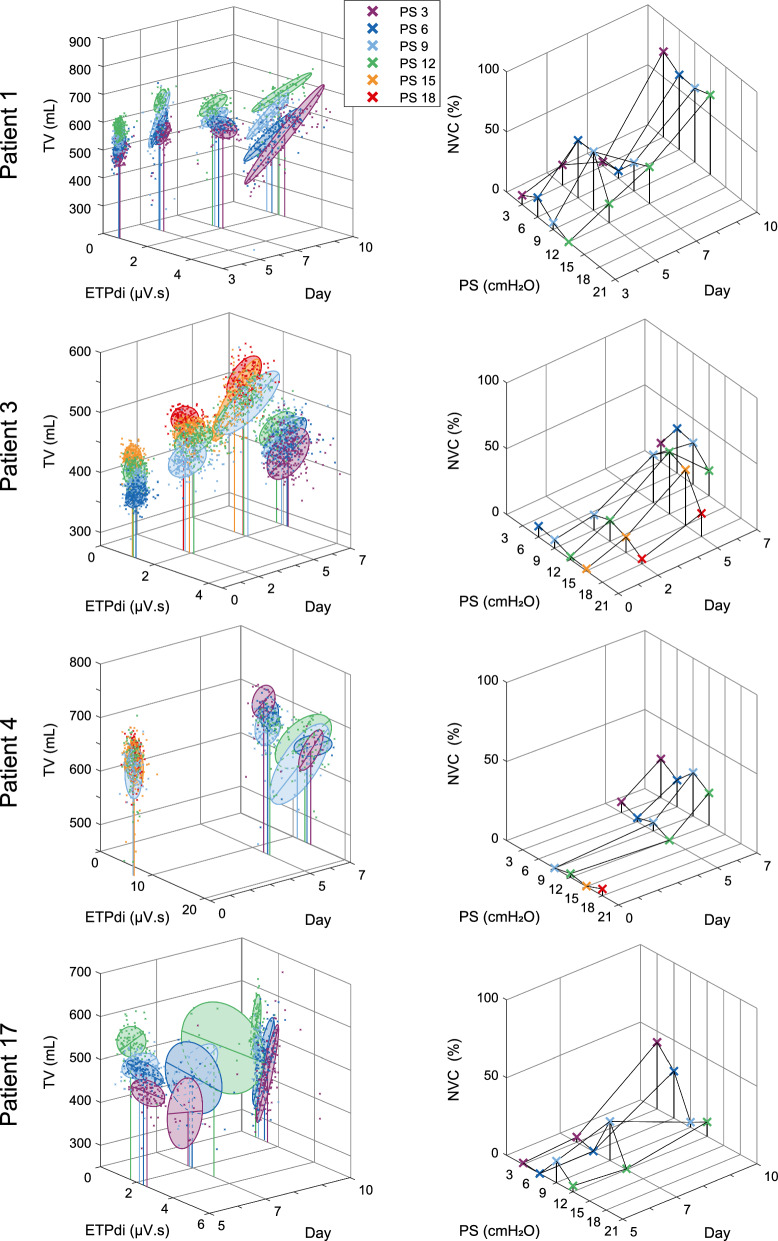
Longitudinal development of breath-by-breath neuro-ventilatory response—Left: ETPdi-TV relation over the days of PSV. Markers are coloured according to the applied PS-level. The ellipses indicate the bivariate distribution of datapoints, whereas the bold line indicates the principal axis of variation. Right: The explained variance by the neuro-ventilatory correlation (NVC) per PS-level over the days of PSV

For longitudinal analysis, valid breath-by-breath data from three or more PS-trials were available in four patients. The development of the NVC during multiple days for these patients is shown in Fig. 5. The data of patient 12 was not included in the longitudinal analysis, as for one PS-trial the number of valid breaths dropped below 10% of the total number of flow-based breaths after matching the neural breaths to flow-based breaths. The NVC showed a trend across two dimensions: the applied support level within a PS-trial, and the day of PSV. Data points were highly clustered, showing a quasi-random distribution, at the more extreme PS-levels and during the earlier days of PSV, whereas the coupling strengthened on later measurement days. The median NVC per PS-trial increased from 3.4% (IQR 2.9) during the first PS-trial to 22.6% (IQR 16.1) at the last PS-trial. The right panels of Fig. 3 illustrate this pattern for an individual patient between the first and last PS-trial. The increase in NVC in these two dimensions was significantly related to higher relative dispersion (IQR/median) of ETPdi values (*R*^2^ = 17%, *p* < 0.01). Within PS-trials, the NVC showed a parabolic pattern with its maximum around the clinically applied PS-levels. The median clinically set PS-level was 8 cmH_2_O (IQR 6–10 cm H_2_O), whereas the maximum was at a median PS-level of 9 cmH_2_O (IQR 6–11.3 cmH_2_O). As patients were ventilated for longer durations, the NVC maximum moved to lower PS-levels. The data of patient 3 followed this trend up to day 5 of PSV, after which the NVC decreased again. Notably, the pulmonary condition of patient 3 deteriorated in the days following the last measurement, requiring reinitiation of controlled MV from which he did not recover. Patients 1, 4 and 17 were weaned successfully.

## Discussion

This study introduces neuro-ventilatory coupling (NVC) as a non-invasive measure to assess an individual patient’s respiratory load capacity. The NVC reflects the breath-by-breath dynamics between NRD and ventilatory output. In contrast, the median values of ETPdi, TV and RR were highly variable and merely indicate NRD or ventilatory output. The strongest NVC was found at the clinically set PS-level, whereas NVC increased when patients approached liberation from MV. These findings indicate the potential of NVC as a bedside parameter to tailor ventilatory support and monitor the patient’s respiratory capacity towards weaning.

### NVC for tailoring ventilatory support

The decrease in NVC in response to both the high and low PS-levels is hypothesized to reflect the transition from a quasi-passive to an active breathing pattern, as introduced by Docci et al. [10]. During overassistance, the patient merely triggers the ventilator and is passively supported thereafter. As TV only partially depends on diaphragm activation, this breathing pattern results in a quasi-random relation between ETPdi and TV, corresponding to small NVCs. During underassistance, the patient makes every effort to meet his ventilatory demands, and breathing variability decreases [17]. As such, the ETPdi and TV uncouple, which is reflected by a decreasing NVC. In the active breathing pattern, on the other hand, the patient makes a greater contribution to the TV, yielding an increasingly higher NVC. As the NVC captures the neuro-ventilatory response to both over- and underassistance, it offers a novel parameter to monitor ventilatory support at the bedside.

### NVC for monitoring respiratory capacity during weaning

The increasing NVC through the course of assisted MV can be attributed to the concurrent processes of neurological, respiratory, and diaphragmatic recovery. As a patient recovers from the critical illness and analgosedation is reduced, diaphragm activity increases [18]. This leads to increased breathing-related variability in ETPdi, as reflected by the larger IQRs of ETPdi in non-sedated patients in this study. The positive correlation between ETPdi variability (IQR/median) and NVC consequently suggests that higher ETPdi variability yields a higher NVC.

The observed increase in NVC might also result from recovery of lung and diaphragm function. Improving lung mechanics requires less driving pressure for the same TV. A recovering diaphragm becomes more efficient, thereby generating more pressure, and thus TV, for the same neural drive. Although the contribution of lung and diaphragm related mechanisms may vary among patients and between underlying diseases, this study suggests that the NVC captures the gradual transition towards an active breathing pattern as patients approach liberation from MV. The NVC thereby offers an accessible bedside measure to quantify respiratory muscle capacity during weaning. This allows for assessment of the relation between ventilatory support, respiratory muscle function and weaning outcomes in ICU patients.

### Variability of averaged ETPdi, TV, and RR limits clinical use

The effect of PS-level on NRD and ventilatory output were evaluated by analysing the average neuro-ventilatory parameters (TV, RR, and ETPdi) per PS-level. The observed large variability of these parameters required normalisation to unmask underlying trends of increasing RR and ETPdi and decreasing TV with decreasing PS-levels. The moderate PS ranges applied in this study, close to the clinical level of support, likely explain the subtle changes in neuro-ventilatory response observed in the context of its variability. Previous studies applied more extreme PS-levels, ranging from 0 to 25 cmH_2_O, and demonstrated significant effects without normalisation [10, 11, 19]. The vast majority of ICU patients is however ventilated in the mid-range of PS settings, as applied in this study. This indicates that average neuro-ventilatory parameters, in contrast to the NVC, exhibit too much variability to monitor bedside respiratory load in individual patients.

Additional caution is warranted regarding the use of averaged neuro-ventilatory parameters, as the coupling between neural drive and ventilatory output changed across PS-levels and over the course of assisted MV. Such underlying mechanisms are masked when only average parameters are considered, whereas they provide valuable insight into the level of ventilatory assistance towards weaning. These findings signify the importance of the NVC, capturing the breath-by-breath relation between neural respiratory drive and ventilatory output.

### Limitations

This study included no reference measures of respiratory effort, such as invasive pressures or occlusion manoeuvres. As a result, only the relation between the NRD and ventilatory output could be assessed through the newly defined NVC. The underlying neuro-muscular response was not examined as done in previous studies [10, 20]. Yet, this approach greatly contributed to the feasibility of this longitudinal study, as well as the future bedside applicability of the NVC, as only sEMGdi and ventilator data are required for its calculation. Although a considerable number of PS-trials were excluded from analysis because of data quality and despite a tolerant exclusion criterion of 10% valid neural breaths, it should be noted that the measurement setup was the same as previously reported [14], having similarly high exclusion rates. By applying the latest recommendations, i.e., using a bilateral sEMG setup, bedside sEMG quality evaluation, and integration of multiple data sources [14, 21], future studies could attain significantly better exclusion rates, ultimately allowing for a stricter neural breath criterion. The longitudinal analysis was performed for only four patients. The patients’ clinical conditions either quickly improved towards extubation or deteriorated towards resumption of controlled MV, resulting into a median of two included PS-trials per patient.

## Conclusion

This study proposes the NVC, a novel parameter to assess the individual patient’s neuro-ventilatory response. The NVC was quantified non-invasively based on advanced, detailed signal analysis of diaphragm sEMG waveforms, and is suggested to reflect the diaphragm’s capacity to overcome mechanical load imposed during assisted MV. A low NVC during higher levels of ventilatory support in the early course of MV, contrasted maximal NVC during moderate PS-levels, with increasing NVC as patients shifted to active breathing and approached liberation from MV. NVC thereby seemingly indicates the diaphragm’s capacity to overcome mechanical load, while the average ETPdi, TV and RR exhibited high variability, limiting their ability to monitor bedside respiratory load in individual patients. NVC creates clinical perspectives for comprehensive monitoring of diaphragmatic load capacity to optimize patient-specific bedside tailoring of ventilatory support towards weaning from MV in ICU patients.

## Supplementary Information

Additional file1

Additional file2

## Data Availability

No datasets were generated or analysed during the current study.

## Abbreviations

sEMG: Surface electromyography
ICU: Intensive care unit
sEMGdi: SEMG of the diaphragm
NRD: Neuro-respiratory drive
PS: Pressure support
TV: Tidal volume
RR: Respiratory rate
ETPdi: Electrical-time-product of sEAdi
NVC: Neuro-ventilatory correlation
R^2^: Explained variance by Pearson correlation coefficient
MV: Mechanical ventilation
CCMO: Central committee on research involving human subjects
FiO2: Fraction of inspired oxygen
SpO2: Oxygen saturation
RASS: Richmond agitation and sedation scale
BMI: Body mass index
ECG: Electrocardiogram
Paw: Ventilator airway pressure
F: Ventilator flow
V: Ventilator volume
ABG: Arterial blood gas
sEAdi: Electrical activity of the diaphragm, envelope of sEMGdi signal
AUB: Area under the baseline
ETPdi: SEAdi time-product
Tbreath: Inter-breath time
IQR: Interquartile range
GEE: Generalised estimating equations
Nincl: Number of included PS-trials
Ntot: Number of acquired PS-trials
PE: Pulmonary embolism
TBI: Traumatic brain injury
COPD: Chronic obstructive pulmonary disease
AAA: Abdominal aortic aneurysm
SSw: Sum of squares within
SStot: Sum of squares total
pp: Percentage point
CoV: Coefficient of variation
PaCO_2_: Arterial partial pressure of CO_2_
PaO_2_: Arterial partial pressure of O_2_
PEEP: Positive end-expiratory pressure
PSV: Pressure support ventilation
