# Supplementary material for: Advanced serial analysis of the diaphragm surface EMG: insights into the effect of pressure support on the neuro-ventilatory response during the ICU stay
Source: Crit Care. 2025 Jun 23;29:258. doi: 10.1186/s13054-025-05424-5 (PMC12186416; doi:10.1186/s13054-025-05424-5)
Supplement: Supplementary file 1 — Additional file1 [file 13054_2025_5424_MOESM1_ESM.docx]

Table 1 - Included and excluded measurements per patient

|  |  | **Pre measurement values** | | | | | | | | **PS trial steps** | **Included** | |
| --- | --- | --- | --- | --- | --- | --- | --- | --- | --- | --- | --- | --- |
| **Subject** | **Day** | **SpO2**  (%) | **FiO2**  (%) | | **RASS**  ( . ) | | **PEEP**  (cmH_2_O) | | **PS**  (cmH_2_O) | -3, +0, +3, +6  (cmH_2_O) | **Median**  **trend** | **Longi-tudinal** |
| 1 | 0 | 94 | 30 | | -1 | | 6 | | 18 | 21, 18, 15, 12 | 0 | 0 |
| 1 | 3 | 98 | 35 | | 0 | | 5 | | 5 | 12, 9, 6, 3 | 1 | 1 |
| 1 | 5 | 99 | 35 | | 0 | | 5 | | 8 | 12, 9, 6, 3 | 1 | 1 |
| 1 | 7 | 99 | 35 | | -1 | | 5 | | 6 | 12, 9, 6, 3 | 1 | 1 |
| 1 | 10 | 99 | 35 | | 0 | | 5 | | 5 | 12, 9, 6, 3 | 1 | 1 |
| 2 | 0 | 100 | 45 | | -2 | | 10 | | 10 | 15, 12, 9, 6 | 0 | 0 |
| 2 | 3 | 100 | 40 | | 0 | | 5 | | 6 | 12, 9, 6, 3 | 0 | 0 |
| 3 | 0 | 94 | 45 | | -2 | | 5 | | 10 | 15, 12, 9, 6 | 1 | 1 |
| 3 | 2 | 96 | 50 | | -4 | | 5 | | 12 | 18, 15, 12, 9 | 1 | 1 |
| 3 | 5 | 92 | 50 | | 0 | | 6 | | 14 | 18, 15, 12, 9 | 1 | 1 |
| 3 | 7 | 95 | 45 | | 0 | | 8 | | 10 | 18, 15, 12, 9 | 1 | 1 |
| 4 | 0 | 97 | 40 | | -4 | | 8 | | 12 | 18, 15, 12, 9 | 1 | 1 |
| 4 | 5 | 96 | 40 | | -4 | | 8 | | 6 | 12, 9, 6, 3 | 1 | 1 |
| 4 | 7 | 95 | 40 | | 0 | | 8 | | 6 | 12, 9, 6, 3 | 1 | 1 |
| 4 | 10 | 98 | 40 | | 0 | | 6 | | 8 | 12, 9, 6, 3 | 0 | 0 |
| 5 | 0 | 96 | 40 | | 0 | | 10 | | 8 | 12, 9, 6, 3 | 1 | 0 |
| 6 | 0 | 93 | 45 | | -4 | | 10 | | 8 | 12, 9, 6, 3 | 1 | 0 |
| 6 | 9 | 96 | 35 | | -4 | | 10 | | 6 | 12, 9, 6, 3 | 0 | 0 |
| 7 | 0 | 99 | 35 | | -3 | | 6 | | 10 | 15, 12, 9, 6 | 0 | 0 |
| 7 | 3 | 97 | 35 | | -1 | | 6 | | 10 | 15, 12, 9, 6 | 0 | 0 |
| 7 | 7 | 99 | 35 | | -1 | | 5 | | 5 | 12, 9, 6, 3 | 1 | 0 |
| 8 | 0 | 96 | 30 | | -3 | | 8 | | 10 | 15, 12, 9, 6 | 0 | 0 |
| 9 | 0 | 100 | 55 | | -4 | | 8 | | 6 | 12, 9, 6, 3 | 0 | 0 |
| 9 | 0 | 100 | 55 | | -4 | | 8 | | 6 | 12, 9, 6, 3 | 0 | 0 |
| 10 | 0 | 94 | 50 | | -5 | | 10 | | 12 | 18, 15, 12, 9 | 1 | 0 |
| 10 | 4 | 95 | 45 | | -4 | | 8 | | 4 | 12, 9, 6, 3 | 1 | 0 |
| 11 | 0 | 90 | 40 | | 0 | | 10 | | 4 | 12, 9, 6, 3 | 1 | 0 |
| 12 | 0 | 99 | 35 | | -4 | | 10 | | 8 | 12, 9, 6, 3 | 1 | 0 |
| 12 | 2 | 98 | 30 | | -2 | | 8 | | 6 | 12, 9, 6, 3 | 1 | 0 |
| 12 | 5 | 99 | 30 | | 0 | | 8 | | 6 | 12, 9, 6, 3 | 1 | 0 |
| 13 | 0 | 99 | 40 | | -1 | | 10 | | 12 | 18, 15, 12, 9 | 1 | 0 |
| 14 | 0 | 99 | 30 | | 0 | | 5 | | 6 | 12, 9, 6, 3 | 1 | 0 |
| 15 | 0 | 95 | 45 | | 0 | | 10 | | 12 | 18, 15, 12, 9 | 0 | 0 |
| 15 | 3 | 95 | 50 | | -3 | | 10 | | 10 | 15, 12, 9, 6 | 0 | 0 |
| 15 | 7 | 94 | 30 | | 0 | | 8 | | 6 | 12, 9, 6, 3 | 0 | 0 |
| 16 | 0 | 94 | 45 | | -2 | | 5 | | 6 | 12, 9, 6, 3 | 1 | 0 |
| 17 | 0 | 95 | 40 | | -4 | | 10 | | 6 | 12, 9, 6, 3 | 0 | 0 |
| 17 | 3 | 94 | 50 | | -4 | | 10 | | 8 | 12, 9, 6, 3 | 0 | 0 |
| 17 | 5 | 99 | 40 | | -2 | | 10 | | 8 | 12, 9, 6, 3 | 1 | 1 |
| 17 | 7 | 100 | 35 | | -2 | | 6 | | 10 | 15, 12, 9, 6 | 1 | 1 |
| 17 | 10 | 100 | 35 | | -1 | | 6 | | 8 | 12, 9, 6, 3 | 1 | 1 |
| 17 | 12 | 100 | 40 | | 0 | | 6 | | 6 | 12, 9, 6, 3 | 0 | 0 |
| 17 | 14 | 99 | 35 | | 0 | | 6 | | 6 | 12, 9, 6, 3 | 0 | 0 |
|  |  |  | |  | |  | | N patients: | | | 13 | 4 |
|  |  |  | |  | |  | |  |  |  | (76%) | (24%) |
|  |  |  | |  | |  | | N datasets: | | | 26 | 14 |
|  |  |  | |  | |  | |  |  |  | (60%) | (33%) |

Abbreviations: SpO2 Oxygen saturation, FiO2 fraction inspired oxygen, RASS Richmond Agitation and Sedation Scale, PEEP positive end-expiratory pressure, PS pressure support
