# Supplementary material for: Advanced serial analysis of the diaphragm surface EMG: insights into the effect of pressure support on the neuro-ventilatory response during the ICU stay
Source: Crit Care. 2025 Jun 23;29:258. doi: 10.1186/s13054-025-05424-5 (PMC12186416; doi:10.1186/s13054-025-05424-5)
Supplement: Supplementary file 2 — Additional file2 [file 13054_2025_5424_MOESM2_ESM.pdf]

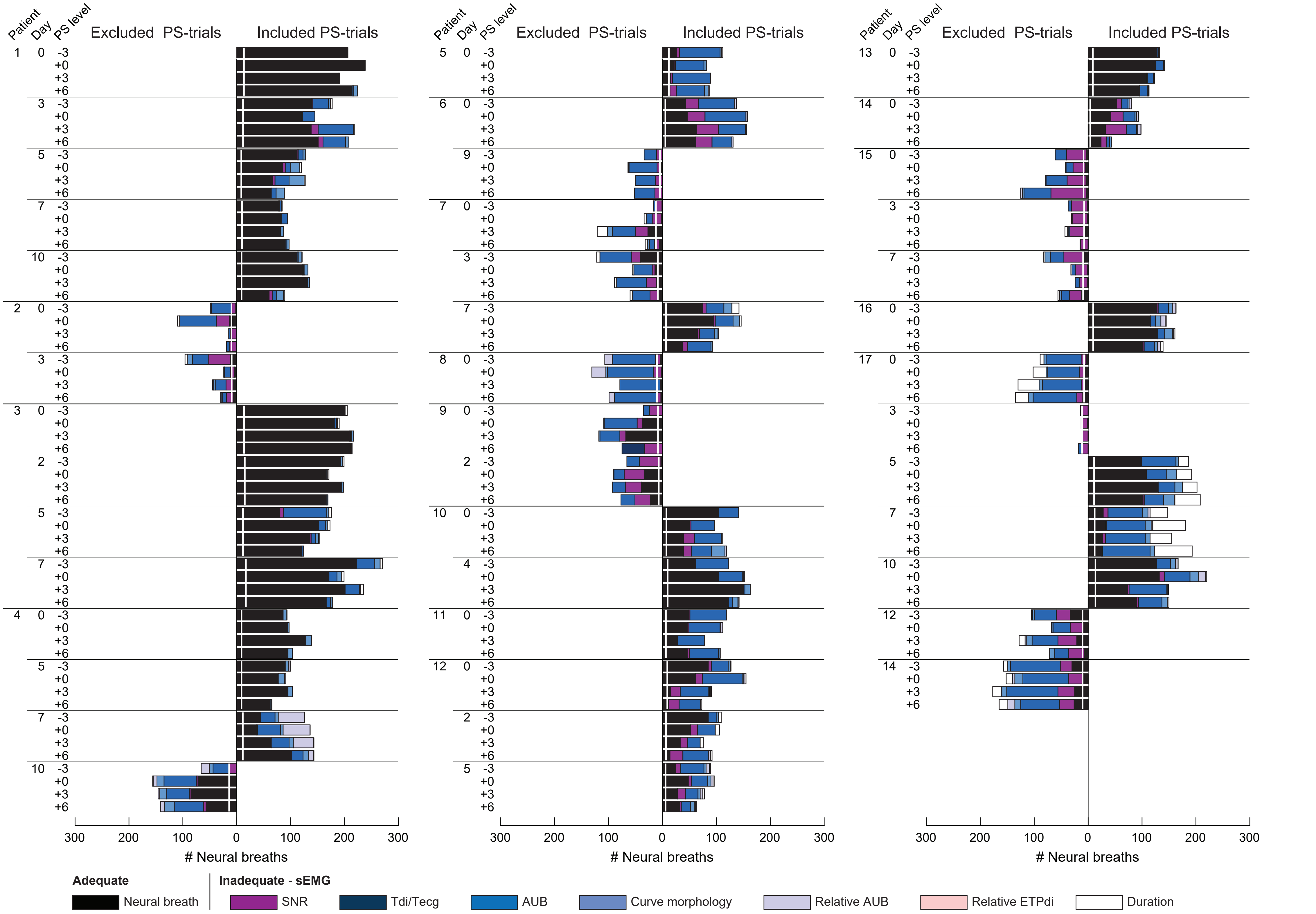

Neural breath permissibility per quality criterion per patient, with individual breaths clustered by whether the PS-trial was in- or excluded according to the quality criteria. Vertical line indicates the 10% of flow-based breaths, as threshold for including a PS-trial. Abbreviations: Adequate neural breath: sEAdi passes all quality checks. SNR – Inadequately low SNR of sEAdi, Tdi/Tecg – Inadequate sEAdi interpeak interval relative to the heart inter-beat interval (ECG), AUB – Inadequately large area under the baseline (AUB), Curve morphology – Inadequate sEAdi due to deviation from bell-morphology, Relative AUB – Inadequately large area under the baseline relative to the whole recording, Relative ETPdi – Inadequately large diaphragm electrical time product (ETPdi) relative to the whole recording, Duration – Inadequately short sEAdi peak duration.
